# Trans‐Generational Morphological Trait Plasticity in Parthenogenetic Offspring of Two *Brachionus dorcas* Morphotypes Induced by *Asplanchna* Kairomones

**DOI:** 10.1002/ece3.72956

**Published:** 2026-01-18

**Authors:** Yali Ge, Jialin Zhu, Yu Ren, Xiaojie Wu, Bo Zhou, Yifan Wu, Yadong Ge

**Affiliations:** ^1^ School of Ecology and Environment Anhui Normal University Wuhu China; ^2^ Collaborative Innovation Center of Recovery and Reconstruction of Degraded Ecosystem in Wanjiang Basin Co‐Funded by Anhui Province and Ministry of Education of the People's Republic of China Anhui Normal University Wuhu China; ^3^ College of Life Sciences Anhui Normal University Wuhu China

**Keywords:** *Asplanchna*, *Brachionus dorcas*, morphological trait, morphotype, parthenogenetic generation, plasticity

## Abstract

We compared trans‐generational (F0–F12) morphological trait plasticity induced by *Asplanchna* kairomones between two *Brachionus dorcas* morphotypes (long‐posterior spines, LS; short‐posterior spines, SS) along with life‐table parameters of the non‐induced morphotypes. Under control conditions, SS rotifers tended to show higher fertility and smaller body size than LS rotifers. Low kairomone concentrations (50 and 200 ind./L) tended to increase body size in SS offspring, while exposure to 50, 200, and 800 ind./L kairomones induced spine elongation in both morphotypes, with posterolateral spine (PS) length increasing with kairomone concentration. Compared to the F0 generation, offspring of both morphotypes in unexposed controls showed generational fluctuations in body size; LS offspring exhibited shortening or no change in anteromedian spine (AMS) and anterolateral spine (ALS) lengths, while SS offspring showed elongation or no change in these spine lengths and PS length. Across all kairomone treatments, significant elongation of AMS and ALS in LS offspring was typically observed only in later generations, whereas SS offspring exhibited significant elongation from F1 through F12; LS offspring showed significant PS elongation from F2 through F12, with maximum lengths in the later generations (F5–F12), while SS offspring showed significant PS elongation from F1 through F12, peaking in early generations (F2–F4). Notably, the multi‐generational mean PS length in SS offspring remained significantly shorter than that in LS offspring under each kairomone treatment. Overall, SS offspring appeared to employ a synergistic defense combining increased body size and spine elongation favoring a “rapid and moderate response,” whereas LS offspring exhibited a “slow and extreme defense” strategy. These divergent strategies may result from evolutionary trade‐offs involving resource allocation, environmental predictability, and genetic constraints.

## Graphical abstract

We compared life‐table parameters and trans‐generation (F0–F12) morphological developmental plasticity induced by *Asplanchna* kairomones between two *Brachionus dorcas* morphotypes (long‐posterior spines, LS; short‐posterior spines, SS). The results revealed that the SS offspring employed a synergistic defense combining increased body size and spine elongation, and favored a “rapid and moderate response,” while the LS offspring exhibited a “slow and extreme defense” strategy, which likely resulted from evolutionary trade‐offs involving resource allocation, environmental predictability, and genetic constraints. This study provides new insights into the evolutionary strategies of adaptive developmental plasticity and the ecological mechanisms of morphological polymorphism in rotifers, and Oikos is the best platform to publish this article.

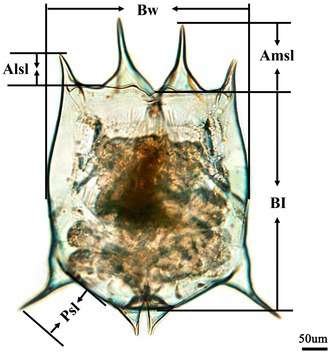

## 1 Introduction

Developmental plasticity, defined as the capacity of a single genotype to produce distinct phenotypes in response to environmental variation, represents a key adaptive strategy enabling organisms to cope with heterogeneous environments (West‐Eberhard 2003; DeWitt and Scheiner 2004; Snell‐Rood and Ehlman 2021). Elucidating its mechanisms has emerged as a central theme in evolutionary biology, developmental biology, and ecology (Hendry 2016; Smallegange 2022; Edwards and Smallegange 2025).

Within natural populations, multiple genotypes coexist. The effects of genotype on specific traits may align with or oppose the direction of environmental effects on developmental plasticity. This interaction can amplify or diminish phenotypic gradients across environments, resulting in genotype‐specific differences in plasticity levels (Sultan and Bazzaz 1993; Conover and Schultz 1995; Thompson 1999; Kelly 2019). Intraspecific differentiations in environmentally induced developmental plasticity constitute a crucial evolutionary strategy for responding to environmental change. As a mechanism of adaptive evolution, this variation may be a key predictor of population dynamics (Kingsolver et al. 2004; Markov and Ivnitsky 2016; Ehrenreich and Pfennig 2016). In‐depth investigation provides essential insights into organismal adaptation mechanisms, extinction risks, and biodiversity maintenance (Forsman 2015; Hendry 2016; Khare et al. 2022).

Theoretical models predict that in species or populations with low genetic variation, highly plastic individuals will exhibit the highest mean fitness under environmental fluctuation suggesting a negative correlation between genetic variation and plasticity (Ahrar et al. 2017; Varela et al. 2021). Rotifers are pivotal components of freshwater ecosystems, serving not only as larval feed but also as primary consumers of algae and bacteria (Herzig 1987; Cavan et al. 2017; McClain and Barry 2010). Monogonont rotifers, such as the cryptic species *Brachionus dorcas* , reproduce both sexually and asexually. Populations generate genetically diverse clones through sexual recombination, while individual clones expand rapidly via parthenogenesis. Parthenogenetic populations have evolved high developmental plasticity as an adaptation to environmental heterogeneity (Bogdan and Gilbert 1982; Gilbert and McPeek 2013; Gilbert 2017). Under heterogeneous conditions, their morphology—particularly traits like body length, width, and spine length—can be directly modified. This morphological diversity significantly enhances their environmental adaptability and population succession capabilities (Rockman and Kruglyak 2006; Ge et al. 2022). Nevertheless, intraspecific variation in morphological developmental plasticity within rotifer species remains poorly understood.

**FIGURE 1:**
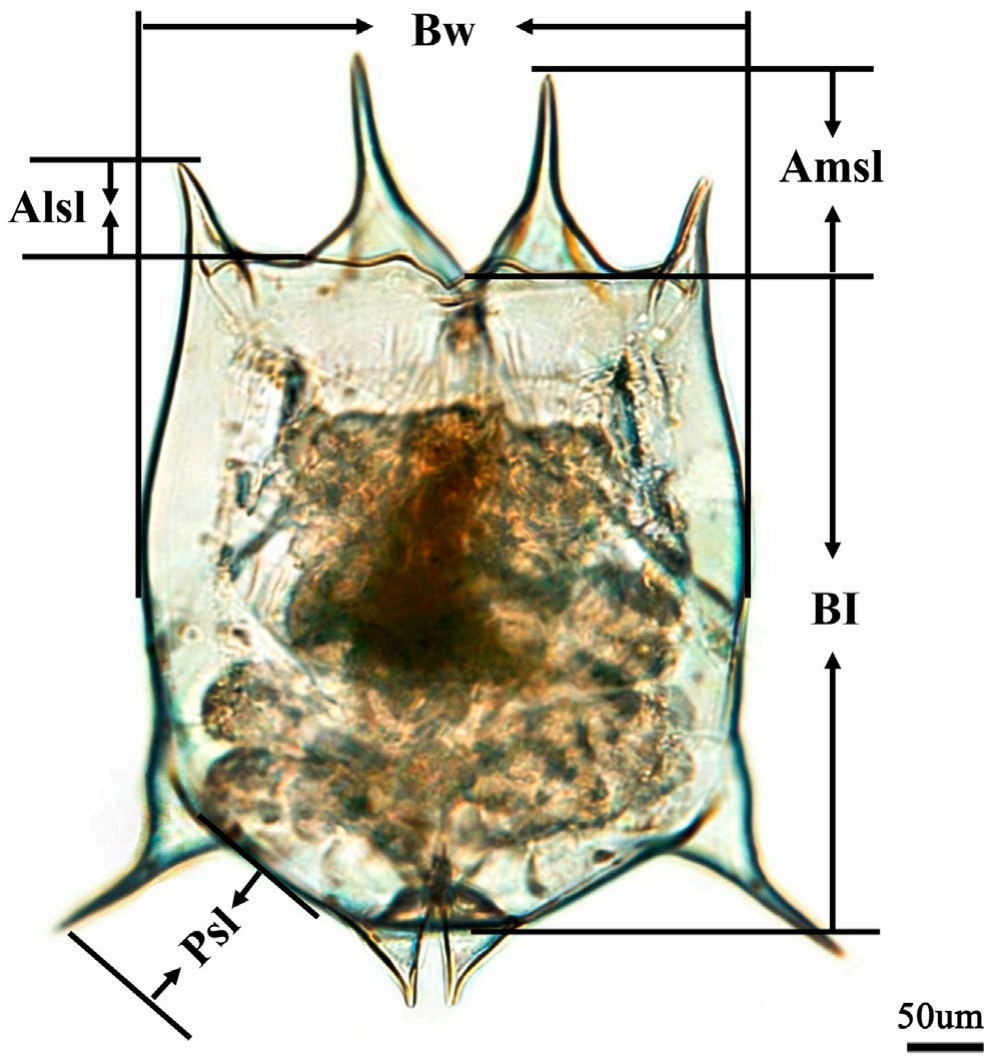
Morphometric parameters of *Brachionus dorcas*. B1: Lorica length; Bw: Lorica width; Ams1: Anteromedian spine length; Als1: Anterolateral spine length; and Ps1: Posterolateral spine length.

*B. dorcas* is commonly found in nutrient‐rich freshwater bodies and serves as a model organism for studying predator‐induced morphological plasticity (Xu et al. 2024; Gilbert 2017). Generally, *B. dorcas* individuals possess two anteromedian spines (AMSs), two anterolateral spines (ALSs), two posterolateral spines (PSs), and two posteromedian spines (Figure 1). *Asplanchna* are predatory rotifers, and their diet predominantly comprises various prey species including ciliates, rotifers, cladocerans, and sometimes even copepods (Arndt 1993), making them an important model organism for laboratory studies of predation (Williamson 1983). Kairomones released by *Asplanchna* can act on the oocytes of *B. dorcas* , inducing the development of effective defensive morphs in their offspring, such as increased body size, a thickened lorica, and elongated spines (Hoverman and Relyea 2009; Gilbert 2012, 2014, 2017; Yin et al. 2015, 2017). Compared to the basic morphotype, these predator‐induced defensive morphologies significantly reduce the risk of detection, capture, and consumption by predators (Iyer and Rao 1996). However, such defenses are argued to confer adaptive advantages only under predation pressure; in predator‐free environments, they may impose fitness costs due to energy expenditure or constraints on mobility (Gilbert 2013). Therefore, we hypothesize that in response to predation pressure, *B. dorcas* clones exhibiting variation in their basic morphology may adopt divergent defensive strategies through energy allocation trade‐offs, facilitating multi‐clonal coexistence.

Although predator‐induced plasticity in rotifers is well‐documented, intraspecific variation in this plasticity—particularly among clones with distinct constitutive morphologies—remains poorly understood. Understanding how different genotypes within a species employ divergent plastic strategies is crucial for elucidating the mechanisms of multi‐clonal coexistence and adaptive evolution in heterogeneous environments. To explore the intraspecific variations in developmental plasticity in rotifers, we established two long‐posterior spine (LS) clones and three short‐posterior spine (SS) clones of *B. dorcas* from resting eggs. This study aimed to: (1) compare the life‐history traits between the non‐induced LS and SS rotifers under non‐inductive conditions; (2) investigate and compare their trans‐generational morphological plasticity in body size and relative spine length (standardized as spine‐to‐body length ratios, S/B ratios—Gilbert 2001) induced by *Asplanchna* kairomones; and (3) elucidate the divergent defensive strategies that may underpin their ecological coexistence. We tested the following hypotheses: (1) SS rotifers would exhibit faster plasticity (i.e., earlier onset of spine elongation) in response to predation risk compared to LS rotifers; (2) due to genetic constraints, the maximum induced spine length in SS rotifers would remain shorter than in LS rotifers; and (3) these differences reflect evolutionary trade‐offs in resource allocation between constitutive defense and inducible plasticity. Overall, this study aims to provide new insights into the potential evolutionary strategies of adaptive developmental plasticity and the ecological mechanisms of morphological polymorphism in rotifers.

## 2 Material and Methods

### 2.1 Rotifer Collection and Culture

Lake Jing (31.33° N, 118.37° E) is a subtropical lake located in Wuhu City, Anhui Province, China. It has a surface area of approximately 0.15 km^2^, an average depth of 1.5 m, and an annual water temperature range of 5.0°C to 36.5°C. *B. dorcas* and *Asplanchna brightwelli* individuals were collected from Lake Jing, and *B. dorcas* was confirmed by molecular phylogenetics based on nuclear internal transcribed spacer (nuITS) sequences (Wen et al. 2016; Xiang et al. 2011). Both rotifer species were subsequently cultured in EPA medium at 25°C ± 1°C under an illumination intensity of approximately 130 lux and a 16 h:8 h light:dark photoperiod.

*B. dorcas* were fed daily the abundant food of green alga *Tetradesmus obliquus* at a density of 2.0 × 10^6^ cells/mL. The algae were cultured in HB‐4 medium (Li et al. 1959), harvested by centrifugation (4000 rpm for 15 min), and stored at 4°C. Algal density was determined using a hemocytometer (Hirschmann, Germany) under a dissecting microscope (Olympus BX61, China). Resting eggs produced during the culture period were collected and stored at 4°C. After 1 month, resting eggs were hatched in EPA medium at 25°C ± 1°C. Individual stem females were then isolated into separate glass zooplankton culture cups and cultured under the conditions described above. By comparing PS lengths of stem females and their parthenogenetic offspring, five robust rotifer clones were selected for further experimentation. Stem females and offspring from clones L1 and L2 consistently exhibited long relative PS lengths and were designated as the long‐spined morphotype (LS). Conversely, stem females and offspring from clones S1, S2, and S3 consistently exhibited short PS lengths and were designated as the short‐spined morphotype (SS).

*A. brightwelli* were fed daily the live prey *B. dorcas* at 10 ind./mL, coinciding with complete medium renewal. Population density was dynamically regulated through daily removal of some individuals to maintain continuous exponential growth phase.

### 2.2 Life‐Table Experiment

Prior to experiments, five clones of *B. dorcas* were pre‐cultured at 25°C for one week. *T. obliquus* (2.0 × 10^6^ cells/mL) was fed and the culture media were changed daily. The rotifer population was maintained in the exponential growth phase by enlarging the total culture volume daily. Life‐table experiments were conducted in 5 mL plastic wells containing 3 mL of medium. For each clone, approximately 200 rotifers carrying amictic eggs were randomly collected and pooled in a 5 mL glass well. Egg hatching was examined under a dissecting microscope every 2 h, and newly hatched neonates (< 4 h old) were used for experiments. Ten neonates were placed in each well. Neonates were cultured under the same conditions described above and observed every 12 h to count the number of living cohorts and the number of newly hatched neonates. After observation, newly hatched neonates were discarded. Culture media were renewed and fresh algae were added every 24 h until all cohorts died. Tests for each clone were repeated three times using 30 individuals independently. Age‐specific survivorship (*l*_*x*_) and fecundity (*m*_*x*_) were constructed for each cohort using conventional life‐table techniques (Poole 1974), and the life‐table demographic parameters, including average lifespan (LS), generation time (*T*), net reproductive rate (*R*_0_), and intrinsic rate of population increase (*r*_*m*_), were calculated using the following formulas (Krebs 1985; Pianka 1988).

$$\mathrm{Net}\;\text{reproductive rate}\;\left(R_{0}\right)=\sum_{0}^{\infty }l_{x}m_{x}$$

$$\text{Generation time}\;\left(T\;\right)=\;\frac{\sum l_{x}m_{x}x}{R_{0}}$$

Intrinsic rate of population increase (*r*_*m*_), was firstly evaluated using: $r_{-\text{rough}}=\frac{\ln R_{0}}{T}$.

For final calculation, we solved the equation: $\sum_{x=0}^{n}e^{-\mathrm{rx}}l_{x}m_{x}=1$.

### 2.3 Trans‐Generation Morphological Response of *B. dorcas* to Predator Kairomones

For kairomone extraction, 800 adult *A. brightwelli* were starved in 1 L EPA medium for 24 h under identical culture conditions. Subsequent processing involved two‐stage filtration through 30‐μm nylon mesh to remove rotifers and large particulates, yielding the predator‐conditioned medium with a nominal kairomone concentration of 800 ind./L. Serial dilutions prepared by volumetric dilution (1:4 and 1:16 ratios) generated sub‐concentrations of 200 and 50 ind./L, respectively. Predator‐conditioned medium was freshly prepared as needed to minimize chemical degradation.

For each *B. dorcas* clone, 125 individuals carrying amictic eggs (F0 generation) were randomly collected. Of these, 25 individuals were fixed in 4% formaldehyde solution for morphological analysis. The remaining 100 individuals were divided into four experimental groups (*n* = 25 per group) and exposed to predator‐conditioned medium in small glass beakers at one of four concentrations: 0 (control), 50, 200, or 800 individuals/L. The exposure volume was 8 mL per beaker, with all other culture conditions as described above. Subsequently, at each kairomone concentration, a total of 75 F1 neonates (< 4 h old) derived from F0 mothers were randomly selected and cultured under conditions identical to the F0 generation. Upon production of the F2 generation, F1 adults were fixed in 4% formaldehyde solution. This sequential exposure and fixation procedure was applied across multiple generations, specifically fixing adult females from generations F0 to F6, F9, and F12. F1 was defined as the earliest induced generation, F2–F4 as earlier induced generations (FE), and F5, F6, F9 and F12 as later induced generations (FL).

Fixed rotifers were examined under a dissecting microscope (Olympus BX61, China), and photos were taken using a digital camera. Lorica length and width, and AMS, ALS and PS lengths were measured using Image J software, with a measurement error of ±0.5%. Rotifer body size (*V*_body_) was calculated as: *V*_body_ = 1/5*a* ^2^ *b* (Zhang and Huang 1991). Relative lengths of anteromedian spine (RAMSL), anterolateral spine (RALSL) and posterolateral spine (RPSL) were calculated as: RAMSL = (*m*_1_ + *m*_2_)/2/*a*, RALSL = (*l*_1_ + *l*_2_)/2/*a*, RPSL = (*p*_1_ + *p*_2_)/2/*a*, respectively; where *a*, *b*, *m*_1_, *m*_2_, *l*_1_, *l*_2_, *p*_1_ and *p*_2_ indicate the lorica length, lorica width, left AMS length, right AMS length, left ALS length, right ALS length, left PS length, and right PS length, respectively.

### 2.4 Data Analyses

Homoscedasticity and normality were assessed using Levene's test and the Kolmogorov–Smirnov test, respectively. A nested one‐way analysis of variance (ANOVA) followed by multiple comparisons was conducted to test the effects of morphotype on life‐table parameters, and a nested three‐way ANOVA was used to evaluate the effects of kairomone concentration, morphotype, and generation number on morphological characteristics (“clone” was nested within “morphotype”). Differences in life‐table parameters and multi‐generational average body sizes and spine lengths among five rotifer clones, differences in morphological parameters among different generations for the same rotifer clone at each kairomone concentration, and differences in multi‐generational average spine lengths among various kairomone concentrations within the same rotifer clone were analyzed using one‐way ANOVA followed by multiple comparisons. The significance level was set at *p* < 0.05. All statistical analyses were conducted in R version 4.4.2.

## 3 Results

### 3.1 Differences in Life‐Table Demographic Parameters Among Five Non‐Induced *B. dorcas* Clones

**FIGURE 2:**
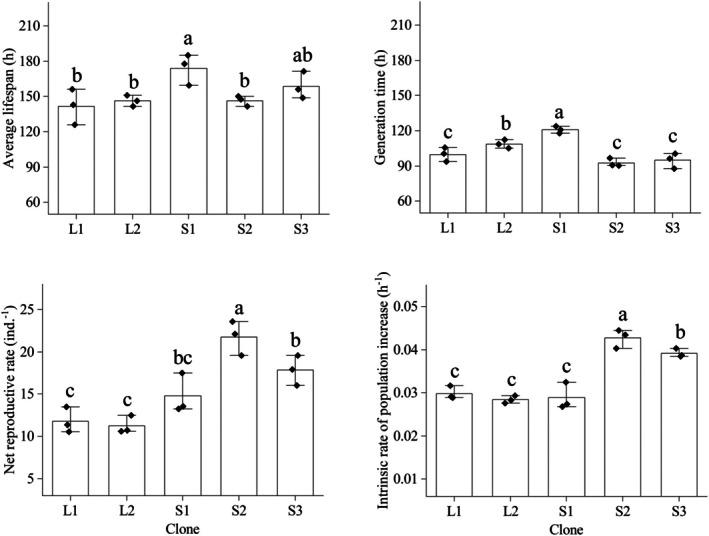
Life‐table parameters of different non‐induced *Brachionus dorcas* clones (mean ± SE). Different letters indicate significant differences among clones (*p* < 0.05); shared letters denote non‐significant differences.

Clone significantly affected all life‐table parameters (*p* < 0.05). SS rotifers tended to exhibit higher fertility and smaller body size than LS rotifers. The average lifespan of S1 was significantly longer than those of L1, L2, and S2, but not S3. Generation time was longest for S1, followed by L2; L1, S2, and S3 had the shortest generation times, with no significant differences among them. Net reproductive rate was the highest for S2, followed by S1; L1 and L2 were intermediate. S3 had a significantly higher net reproductive rate than both L1 and L2, but did not differ significantly from S1 or S2. Intrinsic rate of population increase was the highest for S2, followed by S3; L1, L2, and S1 had the lowest intrinsic rates, with no significant differences among them (Figure 2).

Nested one‐way ANOVA (Table 1) indicated that mean lifespan, net reproductive rate, and intrinsic rate of population increase were significantly influenced by morphotype (*p* < 0.05), but generation time was not (*p* = 0.561).

**TABLE 1:** Effects of morphology on the life‐table demographic parameters of *Brachionus dorcas* .

Parameters	Source	df	SS	MS	*F* value	*p*
Average lifespan	Morphology	1	891.1	891.1	7.787	0.019
	Residuals	10	1144.3	114.4		
Generation time	Morphology	1	8.2	8.2	0.361	0.561
	Residuals	10	226.9	22.7		
Net reproductive rate	Morphology	1	156.26	156.26	48.259	< 0.001
	Residuals	10	32.38	3.24		
Intrinsic rate of population increase	Morphology	1	2.180 × 10^−4^	2.180 × 10^−4^	57.59	< 0.001
	Residuals	10	3.786 × 10^−5^	3.790 × 10^−6^		

Abbreviations: df, degrees of freedom; MS, mean square; SS, sum of square.

### 3.2 Effects of Morphotype, Generation Number and Kairomone Concentration on Body Size in *B. dorcas*

**FIGURE 3:**
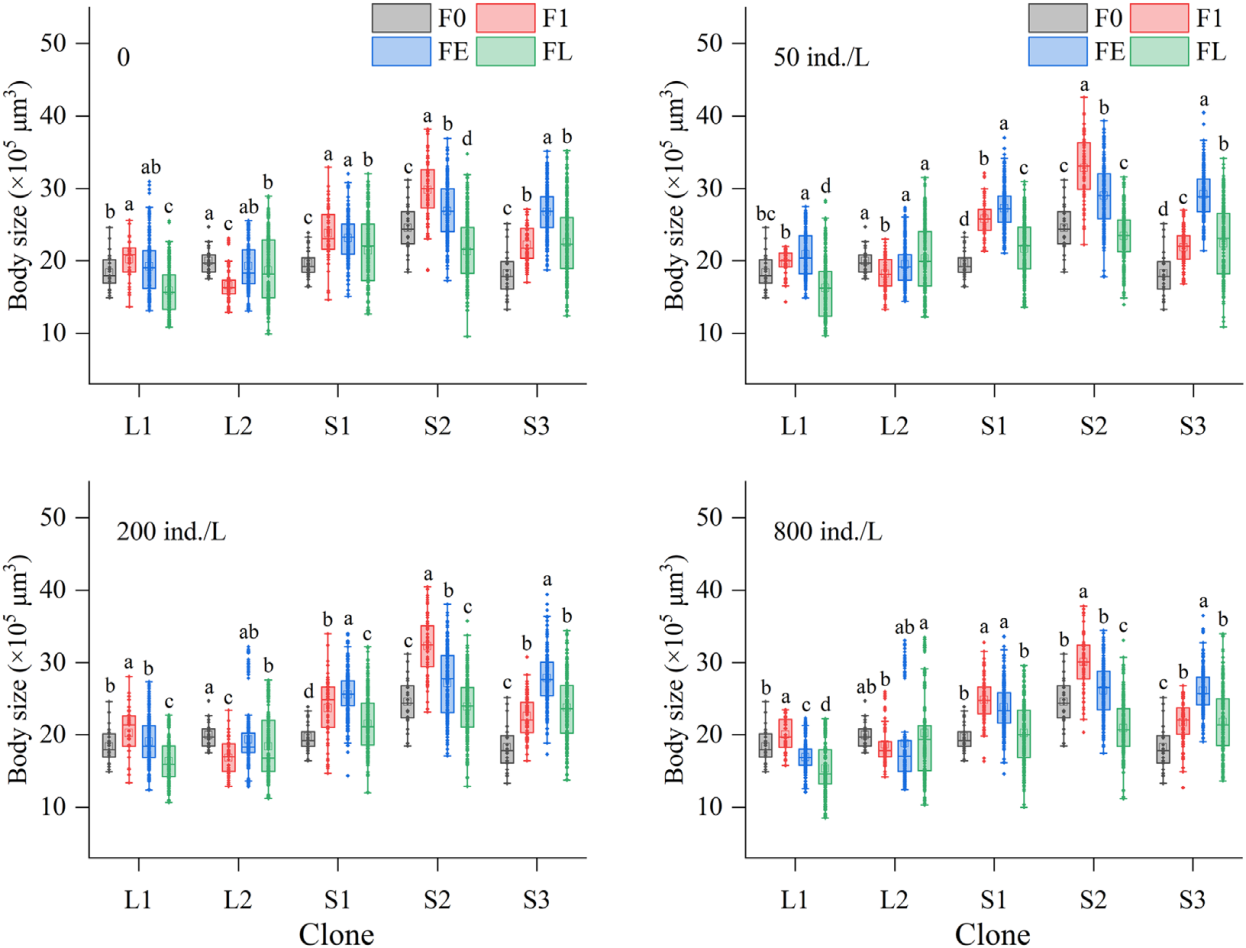
Body sizes of five *Brachionus dorcas* clones in different parthenogenetic generations at four kairomone concentrations (mean ± SE). Different letters indicate significant differences among generations (*p* < 0.05); shared letters denote non‐significant differences.

**FIGURE 4:**
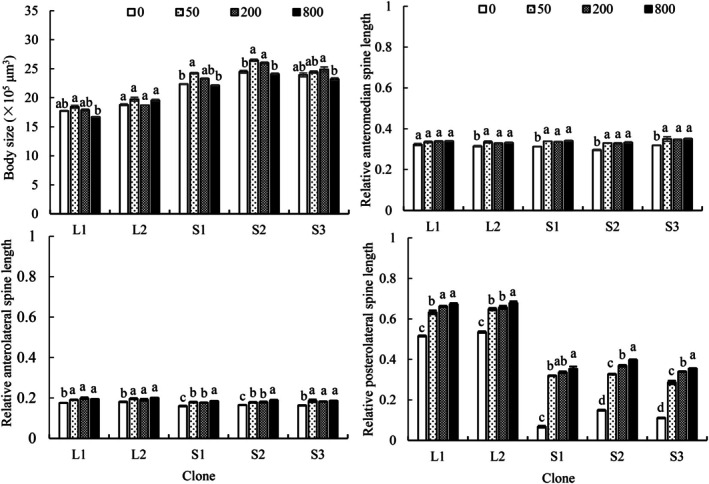
Morphometric parameters of five *Brachionus dorcas* clones at four kairomone concentrations (mean ± SE). Different letters indicate significant differences among various kairomone concentrations (*p* < 0.05), while shared letters denote non‐significant differences.

Body sizes of all five clones in both control and all kairomone treatment groups fluctuated across generations (Figure 3). Clone significantly influenced the multi‐generational mean body size across all kairomone concentrations (*p* < 0.001). SS clones consistently maintained significantly larger body sizes than LS clones throughout the experiment. Kairomone concentration significantly affected mean body size within each clone (*p* < 0.05). Compared to the control, S1 and S2 exhibited higher multi‐generational average body sizes at 50 or 200 ind./L kairomone concentrations but showed no change at 800 ind./L. In contrast, L1, L2 and S3 showed no change in body sizes across the tested kairomone concentrations (Figure 4).

Three‐way nested ANOVA revealed significant effects of generation number, kairomone concentration, morphotype, and all two‐way and three‐way interactions on rotifer body size (*p* < 0.001). Morphotype had the strongest influence (Partial *η* ^2^ = 0.26), with SS clones exhibiting a significantly larger body size than LS clones (Table 2).

**TABLE 2:** The effects of generation number, kairomone concentration and morphology on the morphometric parameters of *Brachionus dorcas* (three‐way (nested) ANOVA).

Parameters	Source	df	SS	MS	*F* value	*p*	Partial *η* ^2^
Body size	Generation number (A)	8	57,564	7195	522.94	< 0.001	0.1555
	Kairomone concentration (B)	3	4723	1574	114.41	< 0.001	0.0128
	Morphology (C)	1	97,302	97,302	7071.59	< 0.001	0.2628
	A × B	24	11,165	465	33.81	< 0.001	0.0302
	A × C	8	10,012	1252	90.95	< 0.001	0.0270
	B × C	3	914	305	22.15	< 0.001	0.0025
	A × B × C	24	7365	307	22.30	< 0.001	0.0199
	Residuals	12,425	170,962	14			0.4618
Relative anteromedian spine length	Generation number (A)	8	5.999	0.7499	363.567	< 0.001	0.1644
	Kairomone concentration (B)	3	1.473	0.4911	238.104	< 0.001	0.0404
	Morphology (C)	1	0.011	0.0106	5.147	0.023	0.0003
	A × B	24	0.898	0.0374	18.134	< 0.001	0.0246
	A × C	8	1.332	0.1665	80.744	< 0.001	0.0365
	B × C	3	0.101	0.0338	16.379	< 0.001	0.0028
	A × B × C	24	0.646	0.0269	13.057	< 0.001	0.0177
	Residuals	12,425	25.628	0.0021			0.7024
Relative anterolateral spine length	Generation number (A)	8	1.183	0.1479	127.755	< 0.001	0.0662
	Kairomone concentration (B)	3	0.857	0.2855	246.669	< 0.001	0.0479
	Morphology (C)	1	0.651	0.6514	562.709	< 0.001	0.0365
	A × B	24	0.382	0.0159	13.756	< 0.001	0.0214
	A × C	8	0.207	0.0259	22.354	< 0.001	0.0116
	B × C	3	0.005	0.0017	1.486	0.216	0.0003
	A × B × C	24	0.174	0.0073	6.278	< 0.001	0.0098
	Residuals	12,425	14.383	0.0012			0.8050
Relative posterolateral spine length	Generation number (A)	8	59.8	7.5	1295.52	< 0.001	0.0932
	Kairomone concentration (B)	3	90.1	30.0	5208.64	< 0.001	0.1405
	Morphology (C)	1	380.8	380.8	66,027.55	< 0.001	0.5939
	A × B	24	17.1	0.7	123.51	< 0.001	0.0267
	A × C	8	8.8	1.1	189.93	< 0.001	0.0137
	B × C	3	3.8	1.3	217.62	< 0.001	0.0059
	A × B × C	24	6.4	0.3	46.22	< 0.001	0.0100
	Residuals	12,425	71.7	0.0			0.1118

Abbreviations: df, degrees of freedom; MS, mean square; SS, sum of square.

### 3.3 Effects of Morphotype, Generation Number and Kairomone Concentration on the Spine Length of *B. dorcas*

**FIGURE 5:**
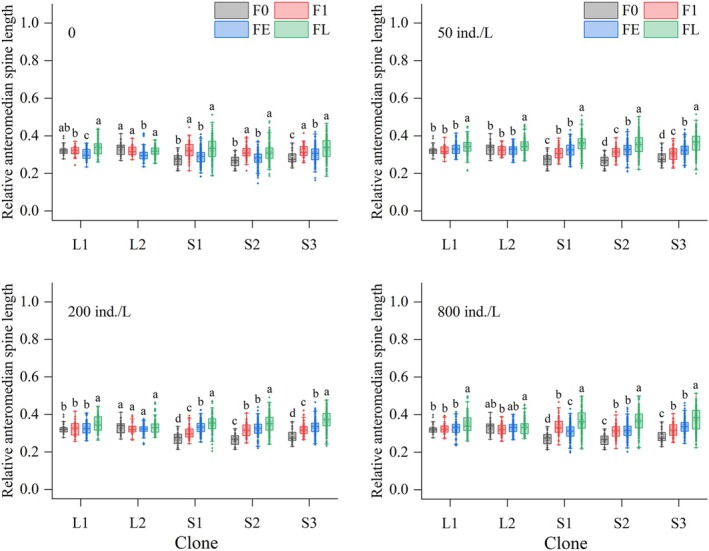
Relative anteromedian spine lengths of five *Brachionus dorcas* clones in different parthenogenetic generations at four kairomone concentrations (mean ± SE). Different letters indicate significant differences among various generations (*p* < 0.05), while shared letters denote non‐significant differences.

**FIGURE 6:**
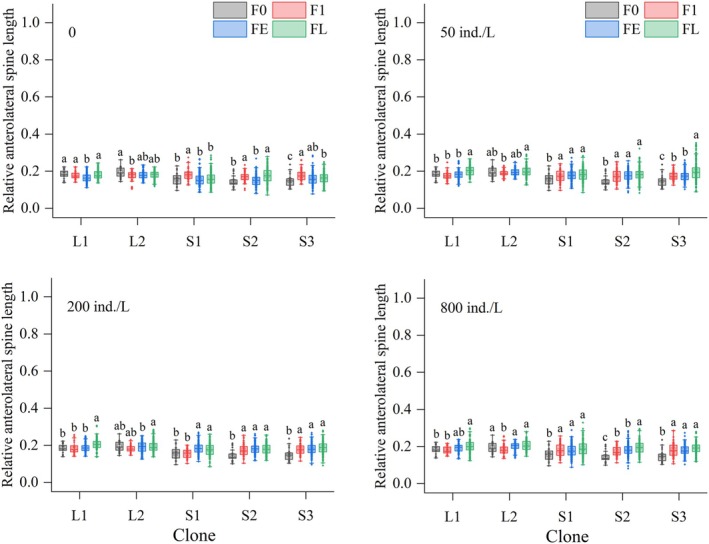
Relative anterolateral spine lengths of five *Brachionus dorcas* clones in different parthenogenetic generations at four kairomone concentrations (mean ± SE). Different letters indicate significant differences among various generations (*p* < 0.05), while shared letters denote non‐significant differences.

**FIGURE 7:**
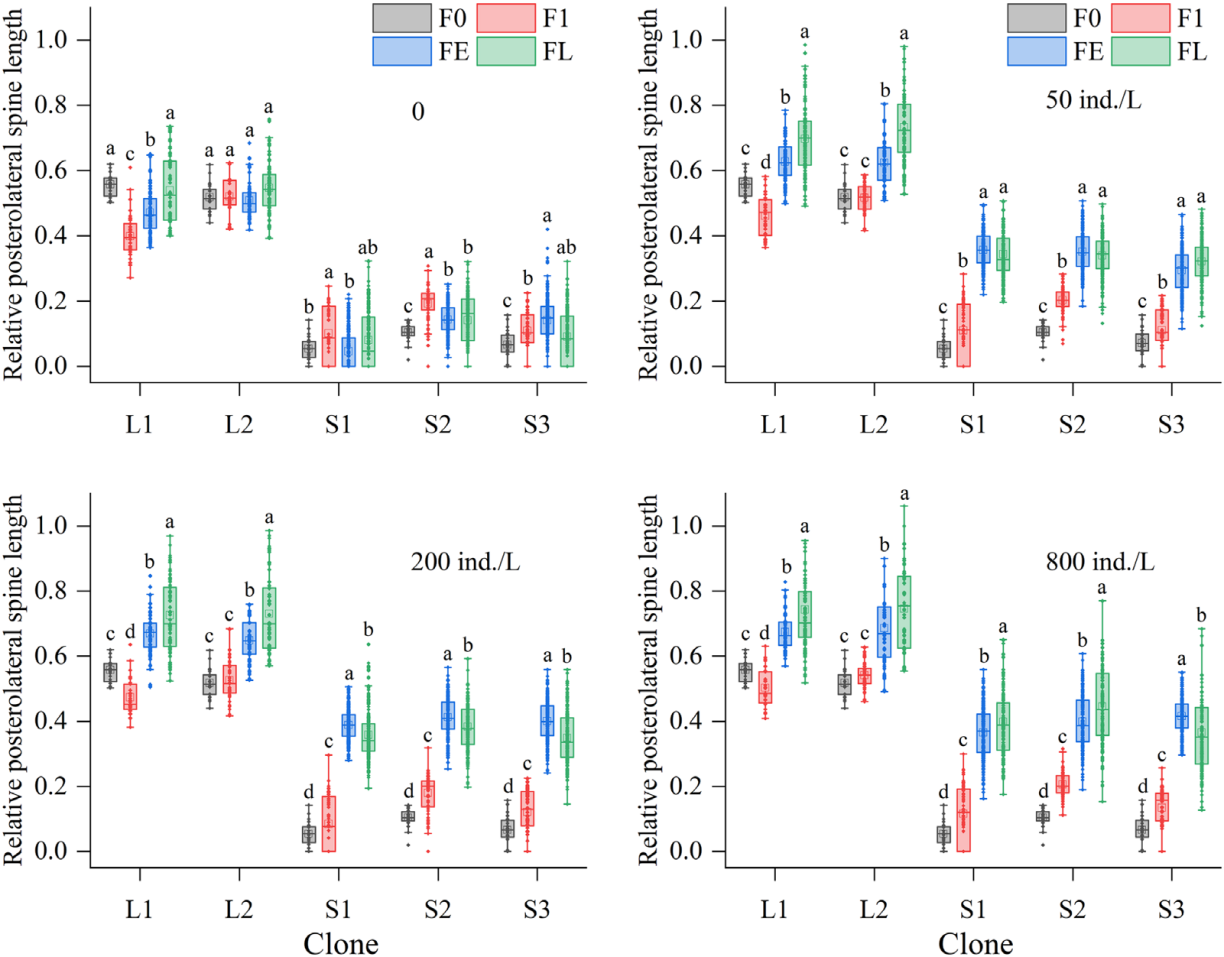
Relative posterolateral spine lengths of five *Brachionus dorcas* clones in different parthenogenetic generations at four kairomone concentrations (mean ± SE). Different letters indicate significant differences among various generations (*p* < 0.05); shared letters denote non‐significant differences.

Across all kairomone treatments, spine lengths in all clones were significantly affected by generation number (*p* < 0.05). For LS clones, compared to the F0, in the control group, AMS and ALS lengths of L1 and L2 were significantly shorter in FE generations, while PS length fluctuated slightly or remained stable. In the 50 ind./L treatment, AMS lengths of both L1 and L2 and ALS length of L1 were significantly elongated in FL generations; ALS length of L2 showed no significant changes; PS length of both L1 and L2 was significantly elongated starting from FE generations, while the magnitude increased over subsequent generations. In 200 and 800 ind./L treatments, AMS and ALS lengths of L1 were significantly elongated in FL generations, while no elongation occurred in L2 offspring; PS length of both L1 and L2 was significantly elongated from the FE generations, reaching maximum values in FL generations (Figures 5, 6, 7).

For SS clones in the control, AMS, ALS, and PS lengths of S1, S2, and S3 significantly increased or remained stable over generations. In the 50, 200, and 800 ind./L treatments, all spine lengths of all three clones were significantly elongated starting from the F1 generation (except ALS length of S1 in the 200 ind./L treatment, which elongated starting from FE generations), peaking in FL generations (Figures 5, 6, 7).

The multi‐generational average spine lengths of all five clones, except AMS length of L1 (*p* = 0.052), were significantly affected by predator kairomone concentration (*p* < 0.05). The average AMS length of L2, S1, S2, and S3, and the average ALS length of L1, L2, and S3 in the control were significantly shorter than observed under each kairomone treatment, with no significant differences among kairomone treatments. The average ALS length of S1 and S2 was shortest in the control, longest at 800 ind./L, and intermediate at 50 and 200 ind./L. For average PS length, *L1* exhibited the shortest value in the control, an intermediate value at 50 ind./L, and the longest values at 200 and 800 ind./L; *L2* and three SS clones exhibited a significant increase in value with increasing kairomone concentration. Notably, SS clones consistently maintained significantly shorter PSs than LS clones (Figure 4).

Three‐way nested ANOVA revealed significant effects of kairomone concentration, generation number, morphotype and their interactions (except the interaction of kairomone concentration × morphotype on ALS length) on spine lengths (*p* < 0.05). Morphotype had the strongest influence on PS length (Partial *η* ^2^ = 0.59), with SS clones exhibiting significantly shorter PS lengths than LS clones (Table 2).

## 4 Discussion

### 4.1 Genetic and Life History Characteristics of Different Morphotypes in *B. dorcas*

Genetic factors are primary endogenous drivers influencing morphological diversity in rotifers (Gilbert 2017). Gilbert and McPeek (2013) demonstrated that in the absence of environmental cues, certain *B. calyciflorus* clones produced female offspring with progressively elongated PS as maternal age increased. This constitutes a bet‐hedging strategy wherein the production of long‐spined offspring ensures that a subset of the population maintains effective anti‐predator defenses, preemptively mitigating predation risk. Our findings reveal that *B. dorcas* can generate clones with divergent spine lengths through sexual reproduction. Under non‐inductive conditions, LS offspring exhibit relatively short spines, whereas SS offspring develop significantly longer spines. This morphological variation stems from genetic differences and represents a further adaptive bet‐hedging strategy, enhancing the population's resilience to fluctuating predation pressures.

The development of elongated spines likely incurs multiple fitness costs. Both constitutively long‐spined and inducible long‐spined morphotypes may exhibit reduced fitness or increased vulnerability to alternative predators (Marinone and Zagarese 1991; Zagarese and Marinone 1992; Gilbert 2013). We found distinct life history traits between the two *B. dorcas* morphotypes under non‐inductive conditions. Non‐induced SS rotifers exhibited a higher reproductive output and larger body size than non‐induced LS rotifers. We speculate that even when not fully induced, long spines, as effective defense structures (Gilbert 2017), may consume more energy and resources for their development and maintenance, leading to a relatively scarce resource available for growth and reproduction of long‐spined individuals (Stearns 1992; Tollrian and Dodson 1999). This differentiation enables adaptation to the spatiotemporal heterogeneity of predation stress in the environment.

### 4.2 Trans‐Generational Morphological Trait Plasticity of Two *B. dorcas* Morphotypes Induced by Predation

#### 4.2.1 Trans‐Generational Body Size Plasticity

Research on zooplankton, such as that by García‐Comas et al. (2016), indicated that increased prey size diversity impeded energy transfer efficiency within food chains, reflecting a trade‐off between individual growth and predator avoidance. In this study, under predator‐free or predator‐present conditions, the body size of both morphotypes in *B. dorcas* exhibited significant trans‐generational variation. Compared to the F0 generation, body size fluctuated across successive generations, with maximum values reaching 1.6–2.0 times the minimum values. This fluctuation may relate to predation avoidance.

The size‐efficiency hypothesis posits that predators exhibit preferences for specific prey sizes (Brooks and Dodson 1965). For instance, visually foraging fish preferentially consume larger prey (Lazzaro 1987; Bremigan and Stein 1994; Nunn et al. 2012), while gape‐limited midge larvae target smaller prey (Swift and Fedorenko 1975; Pastorok 1981). Consequently, prey organisms adopt distinct body size defensive strategies in response to predators with differing size preferences (Nagano et al. 2023). In this study, under predation by *A. brightwellii* , SS offspring exhibited a significant increase or maintained relative stability in body size across parthenogenetic generations under low predation pressure (at kairomone concentration of 50 or 200 ind./L), with increasing multi‐generational average body size. Conversely, under high predation pressure (800 ind./L), their body size fluctuated across generations, and the mean multi‐generational body size was similar to that observed in the absence of predators. In contrast, LS offspring body size fluctuated across generations under all predation intensities, with the multi‐generational mean body size remaining relatively stable or slightly decreasing overall. Therefore, SS offspring displayed body size plasticity under low predation pressure, suggesting that increasing body size may serve as a defense mechanism, consistent with size‐related induced defense phenomenon widely present in cladocerans (Erik et al. 2020). This plasticity was suppressed under high predation pressure, likely due to resource allocation trade‐offs. Under intense predation, SS offspring may prioritize investment in spine elongation—a more effective defense trait (Gilbert 2017)—over further increases in body size, consistent with the theory that phenotypic plasticity incurs costs (Relyea 2002). Conversely, LS offspring lacked active body size adjustment in response to predation, likely because existing spine length provided sufficient defense, allowing reliance on a fixed morphological defense strategy rather than dynamic size changes, a phenomenon also found in other systems where populations adopt different defense strategies due to inherent differences (Van Buskirk 2002).

#### 4.2.2 Trans‐Generational Spine Length Plasticity

In the absence of predator kairomones, AMS, ALS, and PS lengths of LS offspring showed slight shortening in a few generations but remained unchanged in most, aligning with the expectation of reducing unnecessary defense investment to save energy in a safe environment (Tollrian and Dodson 1999). Conversely, SS offspring exhibited slight elongation of these anterior spines in a few generations, with no change in most, which can be regarded as a kind of “preventive” investment, consistent with a “risk‐sharing” strategy for dealing with potential threats (Laforsch and Tollrian 2004). Notably, unlike the anterior spines, PS length of both morphotypes fluctuated or remained stable, suggesting that different defensive traits may be regulated by “modularization,” with differences in development and maintenance costs as well as functional importance (Rabus and Laforsch 2011).

Developmental plasticity enables organisms to acquire optimal morphology by tracking environmental changes. The adaptive benefit depends jointly on its *capacity* (the magnitude of phenotypic adjustment) and its *rate* (speed of response) (Fey et al. 2021; Einum and Burton 2023; Einum and Burton 2025; Burton and Einum 2025). Previous research demonstrated that carnivorous rotifers (*Asplanchna* spp.), cladocerans, and copepods can induce spine development and elongation in *Brachionus* rotifers (Gilbert 2017, 2001; Riessen and Gilbert 2019). Our results confirm that, utilizing developmental plasticity, both morphotypes of *B. dorcas* produced parthenogenetic offspring with elongated spines in response to *A. brightwellii* kairomones. However, trans‐generational plasticity trajectories of spine length differed markedly. In the presence of kairomones, AMS and ALS lengths of LS offspring remained unchanged in early generations (F1–F4) showing significant or a trend towards elongation only in later generations (F5–F12). In stark contrast, SS offspring initiated significant AMS and ALS elongations as early as in the F1, sustaining this response in all subsequent generations, demonstrating a high plasticity *rate* for anterior spine length under predation pressure, rapidly and persistently modifying their morphology. This indicates a high‐priority allocation to inducible defense, sustained even at potentially high metabolic cost. Similar to Gilbert (2001), both morphotypes exhibited significant plasticity in AMS and ALS under *Asplanchna* induction, a malleable defense strategy. We hypothesize that SS offspring have lower basal metabolic costs, freeing metabolic resources for reallocation towards enhanced fecundity, growth and morphological modification, supported by their ability to maintain rapid defensive responses without apparent compromise to reproductive output. This enables rapid phenotypic adjustment to fluctuating predation pressure, as faster plasticity rates can effectively increase environmental predictability (Lande 2014). LS offspring also responded by elongating anterior spines, but their plasticity *rate* was low, characterized by delayed and non‐persistent responses, suggesting relatively conservative defense investment triggered only by sufficiently strong and sustained pressure. We hypothesize that LS offspring allocate energy reserves to maintaining their constitutive long spine length, as evidenced by their reduced reproductive output under non‐inductive conditions, which constrains survival fitness and developmental plasticity. This strategy may reflect an evolutionary adaptation where pre‐emptive investment in a fixed morphological defense reduces reliance on costly plastic adjustments. Under predation pressure, LS offspring opted for slow morphological optimization to minimize short‐term energetic surges. While this constitutive defense strategy with a high plasticity threshold might be advantageous in stable, long‐term predation regimes by minimizing ongoing costs, it inherently reduces adaptive flexibility in rapidly changing environments.

Furthermore, in the present study, under predation pressure, LS and SS *B. dorcas* exhibited significant PS elongation starting from the FE and F1 generation, respectively, sustained in subsequent generations. LS offspring reached maximum PS length in the later generations (F5–F12), while SS offspring attained or approached their maximum length in the early generations (F2–F4). Additionally, across all predation intensities, the multi‐generational mean PS length of SS offspring was significantly shorter than that of LS offspring. Thus, both morphotypes displayed a high plasticity *rate* for PS elongation in response to predation. However, their plasticity *capacities* differed. SS offspring adopted a “pay‐as‐you‐go” strategy, rapidly upregulating the defensive trait (PS length) to a moderate, yet functionally sufficient level upon detecting predator cues. This strategy could be advantageous in environments with highly fluctuating or unpredictable predation pressure, minimizing the cost of maintaining high defenses during safe periods based on their rapid response kinetics and maintained reproductive capacity. If plasticity in a trait is adaptive, the cost of adaptation incurred due to environmental change should be minimized once the phenotype is fully adjusted; therefore, the rate at which the phenotype approaches this state determines the duration of suboptimal phenotype expression and partly dictates the magnitude of fitness costs associated with environmental change (Einum and Burton 2023). In contrast, LS offspring employed a “long‐term investment” strategy. They not only possessed a high baseline defense but also, under sustained predation pressure, continuously invested resources to optimize the defensive structure over multiple generations, potentially reaching physiological or developmental limits, involving an additive effect of genotype and plasticity on PS length (Gilbert 2017). This strategy might be superior in environments with consistently high predation intensity, as maximized defensive capability could confer the highest long‐term survival and fitness.

## 5 Conclusions

Morphological plasticity constitutes a key defensive strategy in rotifers against predation, with obvious energetic costs for developing and maintaining defensive traits. The two *B. dorcas* morphotypes exhibit divergent trans‐generational plastic responses to constant predator threat. The observed strategies—a rapid, moderate response in SS offspring and a slow, maximized response in LS offspring—suggest potential ecological niche differentiation. We speculate that these strategies may reflect adaptations to different predation regimes: the SS strategy could be favored in variable environments, while the LS strategy might prevail under sustained high predation.

## Author Contributions

**Yali Ge:** conceptualization (equal), funding acquisition (lead), methodology (equal), resources (lead), supervision (equal), writing – review and editing (equal). **Jialin Zhu:** data curation (equal), investigation (equal), software (lead). **Yu Ren:** data curation (equal), formal analysis (lead), investigation (equal). **Xiaojie Wu:** data curation (equal), investigation (equal). **Bo Zhou:** data curation (equal), investigation (equal). **Yifan Wu:** data curation (equal), investigation (equal). **Yadong Ge:** conceptualization (equal), methodology (equal), supervision (equal), writing – review and editing (equal).

## Funding

This work was supported by the National Natural Science Foundation of China (32070463) and the Foundation of the University Synergy Innovation Program of Anhui Province (GXXT‐2020‐075).

## Conflicts of Interest

The authors declare no conflicts of interest.

## Data Availability

All data generated or analyzed during this study are included in this published article.
